# Large-Scale Production of Bioactive Terrein by *Aspergillus terreus* Strain S020 Isolated from the Saudi Coast of the Red Sea

**DOI:** 10.3390/biom9090480

**Published:** 2019-09-12

**Authors:** Hani Z. Asfour, Zuhier A. Awan, Alaa A. Bagalagel, Mahmoud A. Elfaky, Reda F. A. Abdelhameed, Sameh S. Elhady

**Affiliations:** 1 Department of Medical Microbiology and Parasitology, Faculty of Medicine, Princess Al-Jawhara Center of Excellence in Research of Hereditary Disorders, King Abdulaziz University, Jeddah 21589, Saudi Arabia; hasfour@kau.edu.sa; 2 Department of Clinical Biochemistry, Faculty of Medicine, King Abdulaziz University, Jeddah 21589, Saudi Arabia; zawan@kau.edu.sa; 3 Department of Clinical Pharmacy, Faculty of Pharmacy, King Abdulaziz University, Jeddah 21589, Saudi Arabia; abagalagel@kau.edu.sa; 4 Department of Natural Products and Alternative Medicine, Faculty of Pharmacy, King Abdulaziz University, Jeddah 21589, Saudi Arabia; melfaky@kau.edu.sa; 5 Department of Pharmacognosy, Faculty of Pharmacy, Suez Canal University, Ismailia 41522, Egypt; omarreda_70@yahoo.com; 6 Department of Pharmacognosy, Faculty of Pharmacy, Port Said University, Port Said 42526, Egypt

**Keywords:** Red Sea, sponges, deep sediment, phylogenetic diversity, *Aspergillus terreus*, terrein, cytotoxic, antimicrobial

## Abstract

The diversity of symbiotic fungi derived from two marine sponges and sediment collected off Obhur, Jeddah (Saudi Arabia), was investigated in the current study. A total of 23 isolates were purified using a culture-dependent approach. Using the morphological properties combined with internal transcribed spacer-rDNA (ITS-rDNA) sequences, 23 fungal strains (in the majority *Penicillium* and *Aspergillus*) were identified from these samples. The biological screening (cytotoxic and antimicrobial activities) of small-scale cultures of these fungi yielded several target fungal strains which produced bioactive secondary metabolites. Amongst these isolates, the crude extract of *Aspergillus* *terreus* strain S020, which was cultured in fermentation static broth, 21 L, for 40 days at room temperature on potato dextrose broth, displayed strong antimicrobial activities against *Pseudomonas aeruginosa* and *Staphylococcus aureus* and significant antiproliferative effects on human carcinoma cells. Chromatographic separation of the crude extract by silica gel column chromatography indicated that the S020 isolate could produce a series of chemical compounds. Among these, pure crystalline terrein was separated with a high yield of 537.26 ± 23.42 g/kg extract, which represents the highest fermentation production of terrein to date. Its chemical structure was elucidated on the basis of high-resolution electrospray ionization mass spectrometry (HRESIMS) or high-resolution mass spectrometry (HRMS), 1D, and 2D NMR spectroscopic analyses and by comparison with reported data. The compound showed strong cytotoxic activity against colorectal carcinoma cells (HCT-116) and hepatocellular carcinoma cells (HepG2), with IC_50_ values of 12.13 and 22.53 µM, respectively. Our study highlights the potential of *A. terreus* strain S020 for the industrial production of bioactive terrein on a large scale and the importance of future investigations of these strains to identify the bioactive leads in these fungal extracts.

## 1. Introduction

The marine habitat is an attractive source of both biological and chemical diversity. It has been reported that oceans contain nearly 291,000 described species, representing only a small number of the total species that have yet to be discovered [1,2,3]. Almost all macro-organisms in marine habitats, e.g., sediment, fish, algae, sponges, corals, ascidians, have been investigated for their content of natural product entities [4,5]. A bioactive compound with unique structures has been isolated and was shown to possess novel anti-inflammatory, antitumor, and antimicrobial properties [6,7,8,9,10,11,12,13,14]. As interests have turned to marine symbionts, fungi have now begun to be recognized as a likely source of bioactive natural products after having received little attention from natural products chemists [15]. Recently, marine-associated fungi have proved to be a gorgeous source of pharmacologically active natural compounds [14,16,17,18,19,20,21,22,23,24,25,26,27]. Most of these micro-organisms grow in a unique, extreme, and stressful environment; therefore, they have the ability to yield unusual and unique secondary metabolites [15].

Fungi belonging to the *Aspergillus* genus are one of the major contributors to the secondary metabolites of fungal origin [14]. Marine-derived isolates of *Aspergillus terreus* are well known for the production of structurally diverse and biologically active natural products [28,29]. Terrein, a fungal metabolite isolated from *A. terreus*, has been proven to perform diverse biological activities [30,31,32,33,34,35]. Although terrein has application value in the fields of medicine, cosmetology, and agriculture, large-scale production of pure (+)-terrein cannot be achieved easily. The production of terrein by chemical synthesis is difficult due to its stereoselectivity [36,37,38,39] and high-cost chemical reagents [40,41]. In this study, we aimed to use fungal fermentation as a method of low-cost production of terrein in high yield. To our knowledge, the present study describes the highest fermentation production of terrein to date [29,42,43,44,45,46,47,48,49].

The static culture broth and mycelia of *A. terreus* S020 fungus were extracted, and the combined extracts were partitioned by silica gel column chromatography. The pure compound terrein was finally purified using a C18 semi-preparative HPLC column.

As part of our ongoing study to isolate and identify compounds from a marine host [50,51,52,53,54], we cultured and identified fungi from a marine source, assessed bioactive activity against *Pseudomona aeruginosa* ATCC27853, *Bacillus* subtilis ATCC6633, S*taphylococcus aureus*, ATCC25923 and *Candida albicans*ATCC76615, and cytotoxic effects on breast adenocarcinoma (MCF-7), hepatocellular carcinoma (HepG2), and colorectal carcinoma (HCT-116) cells, using crude extracts of the cultured fungal isolates. Further separation, structure determination, and bioactive assessment of the yielded metabolites of selected target strains were performed. A compound, terrein, was separated and purified from the static culture broth of *A. terreus* strain S020 (Figures S1 and S2). Its chemical structure was elucidated on the basis of HRMS, 1D, and 2D NMR spectroscopic analysie and by comparison with reported data. The isolated compound was tested for its antiproliferative activity.

## 2. Experimental Section

### 2.1. Biological Materials

The deep-sea sediment and the marine sponge specimens used in this study were collected from the Red Sea at Obhur, Jeddah, Saudi Arabia, in January 2016 by SCUBA diving at depths of 17 and 27 m. After collection, the materials were kept at −25 °C until investigation.

### 2.2. Fungi Isolation

#### 2.2.1. Sponge Samples

Surface sterilization of the sponge samples was performed. The sponge samples were disinfected with 5% sodium hypochlorite, followed by 70% ethanol [55]. The inner tissues of sponge materials were cut into pieces of approximately 2 cm^3^ and homogenized aseptically with sterile, artificial seawater. Three dilutions (1:10, 1:100, and 1:1000) of the resulting homogenate were made with sterile seawater.

#### 2.2.2. Sediment Samples

The deep-sea sediment sample was homogenized aseptically with 15 mL of sterile water, and the resulting solution was diluted by a serial dilution method.

**Figure 1:**
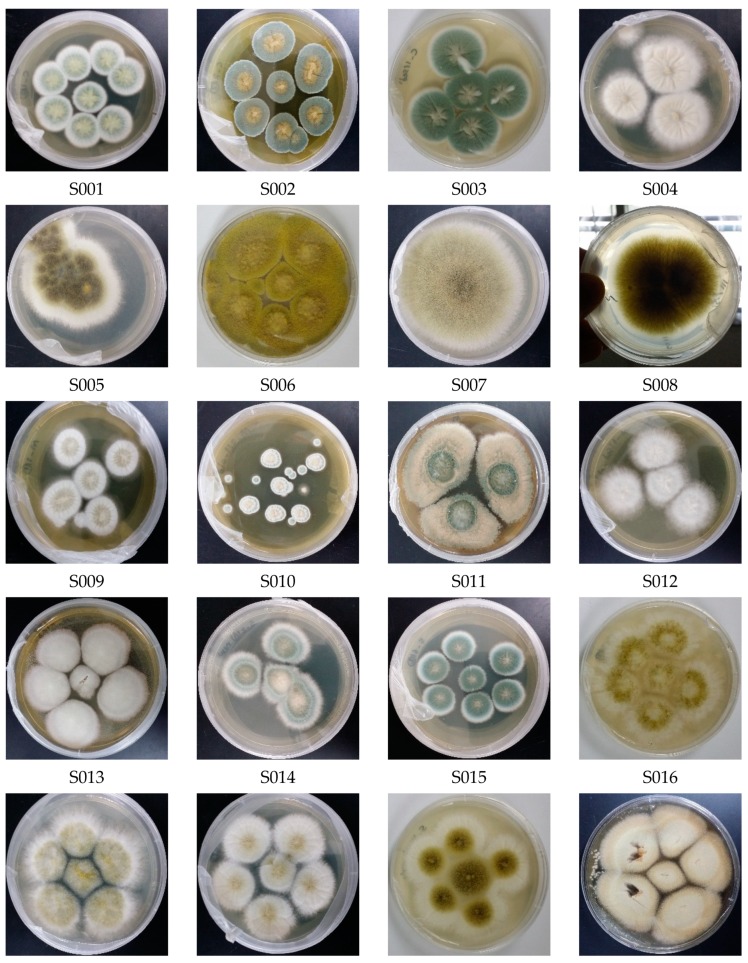

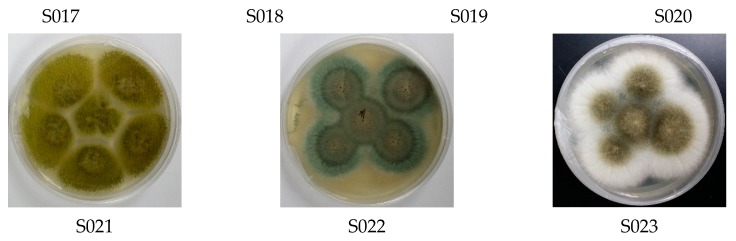
Morphological photos of the 23 derived fungal isolates (S001–S023).

For fungal cultivation, 90 μL of each diluted homogenate was transferred onto plates of each of the following media in triplicate (HiMedia Laboratories, Mumbai, India): Czapek–Dox yeast agar medium (CYE), malt agar medium (ME), and Sabouraud dextrose agar medium (SD). All media were supplemented with an antibiotic (0.25% chloramphenicol) and 2% NaCl. According to Wei’s morphological criteria [56], the fungal isolates were identified morphologically on agar plates after 7–14 days incubation at 29 °C. A series of purification and subculture steps were carried out to obtain purified fungal isolates, and photos were taken of each pure isolate (Figure 1).

### 2.3. DNA Genome Extraction from Pure Subcultured Fungal Isolates

The distinct, pure fungal isolates described above were subcultured in Sabouraud dextrose liquid medium for 3–7 days at 29 °C. The mycelia were separated by filtration and dried using a freeze dryer. According to the manufacturer’s instructions, the fungal DNA extraction of the resulting tissues was performed using the QIAamp DNA Mini Kit (Qiagen, Valencia, CA, USA). The integrity of the extracted DNA was checked and confirmed by gel electrophoresis.

### 2.4. Internal Transcribed Spacer-rDNA (ITS-rDNA) Fragments Amplification, Sequencing, and Phylogenetic Analysis of Fungal Isolates

Using the primers ITS1 (5′-TCCGTAGGTGAACCTGCG-3′) and ITS4 (5′-TCCTCCGCTTATTGATATGC-3′) [57], the genomic DNA of the fungal isolates was used as the template to amplify fungal ITS-rDNA fragments. For preliminary identification, the sequences of fungal ITS-rDNA regions were compared with related sequences at the National Center for Biotechnology Information (NCBI) as previously described [58,59,60].

### 2.5. Fermentation and Preparation of Extracts of the Fungal Isolate S020

*A. terreus* strain S020 was cultured under static conditions at room temperature in 2 L Erlenmeyer flasks containing 500 mL of potato dextrose liquid culture medium (PDB). After 40 days of cultivation, 21 L of whole broth was filtered through cheesecloth to separate the culture broth from the mycelia. The broth was extracted three times with ethyl acetate (EtOAc), while the mycelia were extracted three times with methanol (MeOH). Both EtOAc and MeOH extracts showed a similar TLC pattern and so were combined and concentrated to generate a crude extract (17.49 g) for further separation.

### 2.6. Isolation and Purification of Terrein

The total crude extract (17.49 g) of *A. terreus* strain S020 was subjected to silica gel column chromatography (CC) using gradient elution of *n*-hexane, CHCl_3_, and MeOH at a flow rate of about 20 mL/min. Fractions of 100 mL were collected and examined by TLC; similar fractions were combined and evaporated under reduced pressure to obtain fractions 1–10. Fraction 5 (CHCl_3_ fraction, 13.50 g) was subjected to silica gel CC with CHCl_3_–MeOH gradient elution to afford seven further fractions. Of these, the bioactive fractions eluted with CHCl_3_–MeOH (20:1) were concentrated to yield a terrein precipitate (9.20 g, purity 85%) which was finally purified by HPLC (XDB-C18 Zorbax, 5 µm, 250 mm × 4.6 mm) using 20% CH_3_CN/H_2_O at a flow rate of 1 mL/min and UV detection at 281 nm.

Terrein was characterized as follows: Colorless crystal needles (10.3 mg); $\alpha_{D}^{25}$ + 151.0 (*c* 0.5, MeOH); UV (λ_max_, MeOH) (log ε): 227 (4.31), 281 (2.51) nm; spectroscopic NMR data: (see Supplementary Materials); ESI-MS: *m*/*z* 155.07 [M + H]⁺; high-resolution electrospray ionization mass spectrometry (HRESIMS): *m*/*z* 155.0698 (calculated for C_8_H_11_O_3_ [M + H]⁺, 155.0708).

### 2.7. Biological Activity

#### 2.7.1. Preparation of the Extracts of Isolates S001–S023

The fungal strains S001–S023 were inoculated into 250 mL Erlenmeyer flasks containing 50 mL of the corresponding liquid media (Table 1), incubated at 29 °C, and continuously shaken at 150 rounds per minute (rpm) in an orbital shaker for 14 days. After incubation, 50 mL of EtOAc was added to each flask and left overnight to stop cell growth. The mycelia were separated by filtration, and the filtrate was extracted three times (3 × 50 mL) with EtOAc. The organic portion (combined extracts) was evaporated under vacuum, and the residues obtained were washed with water and then taken to dryness to obtain colored crude extracts (broth extract). Other crude extracts (mycelia extract) were obtained by extraction of mycelia with MeOH and evaporation of the solvent under a vacuum. The resulting EtOAc and MeOH extracts were lyophilized and stored for biological screening.

#### 2.7.2. Antimicrobial and Cytotoxic Activities of Fungal Extracts of Isolates S001–S023

The crude extracts of the broth and mycelial biomass were subjected to antimicrobial activity assessment against four pathogenic microorganisms: *P. aeruginosa* (ATCC27853), *B. subtilis* (ATCC6633), *S. aureus* (ATCC25923) and *C. albicans* (ATCC76615). Assays were performed by placing 50 µL of the test extract solution (2 mg/mL, DMSO) into each hole on the plates and allowing solutions to stand overnight into an incubator at an appropriate temperature. Activity is indicated by the presence of a clear zone of growth inhibition surrounding the holes. Inhibition zones were measured in mm, and the results are reported in Table 1.

The cytotoxic activity of the test extracts on MCF-7, HCT-116, and HepG2 carcinoma cells was tested using the sulforhodamine B (SRB) assay, as previously described in our studies [54], and the results are presented in Table 2.

## 3. Results and Discussion

### 3.1. Diversity of Culturable Fungal Strains Derived from the Marine Samples

The cultivation of fungal strains from two marine sponges (*Stylissa carteri* and *Hyrtios erectus*) and deep-sea sediment of the Red Sea yielded a total of 36 isolates. Based on morphological traits as well as DNA analysis of the ITS regions [57,61], the redundant strains were excluded, and 23 distinct, pure isolates, were identified (S001–S023; Table 3, Figure 1). The strains from *Penicillium* spp. and *Aspergillus* spp. accounted for a large proportion of the total isolates. Twenty-three isolates were identified on the basis of morphological traits at the genus and species levels via genomic DNA extraction and sequencing analysis.

**Figure 2:**
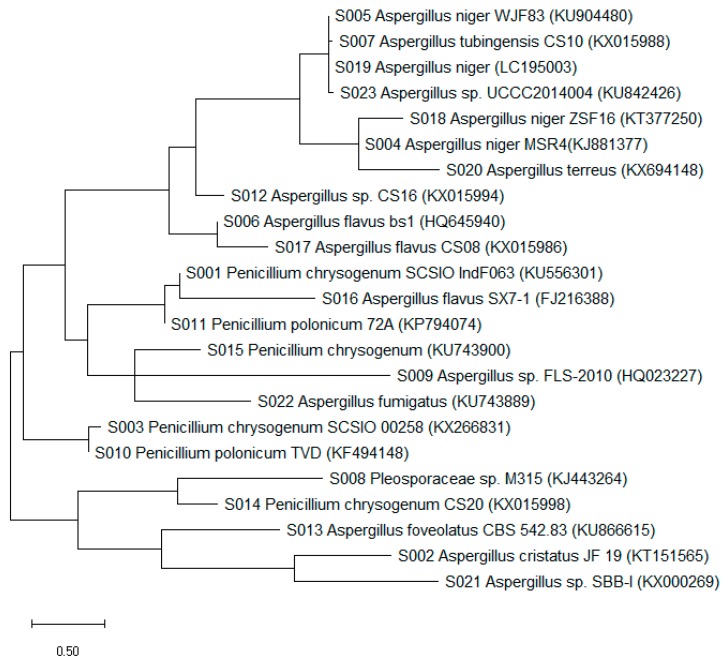
Phylogenetic tree of isolated fungal strains S001–S023.

The phylogenetic tree of fungal strains (Figure 2) represents those fungi that are easily cultivable and could be recovered when culture-dependent techniques are applied [62]. Representative fungal isolates of these strains that have been previously cultured from marine samples include sponges, algae, cnidarians, and sea grasses [63]. The marine invertebrate symbiotic fungi have been reported as a rich source of bioactive secondary metabolites, such as polyketides, with antimicrobial and/or antitumor activities [14,62,63,64]. Our study revealed that the diversity of symbiotic fungal isolates (*Penicillium*, 6 strains; *Aspergillus*, 17 strains; *Pleosporaceae*, 1 strain) obtained from the marine samples was high.

### 3.2. Large-Scale Production and Purification of Terrein from A. terreus Strain S020

The culture broth and mycelia of *A. terreus* strain S020 were extracted using organic solvents, and the combined extracts were fractionated using silica gel column chromatography. The compound terrein was finally purified using a C18 semi-preparative HPLC column.

### 3.3. Structure Elucidation of the Isolated Terrein

**Figure 3:**
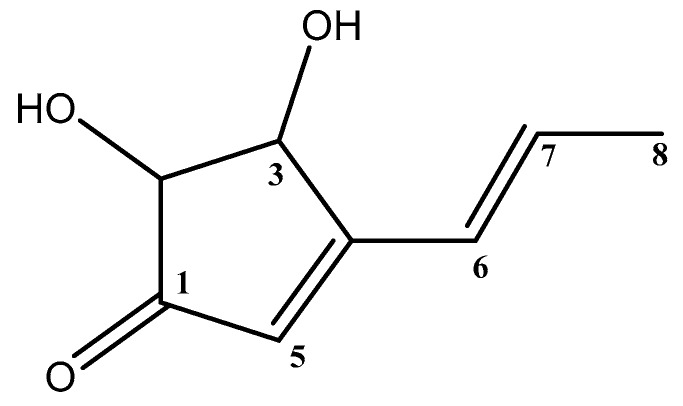
The structure of terrein.

Terrein (Figure 3) was isolated and purified as pale yellow crystal needles. The molecular formula, C_8_H_10_O_3_, was determined to be *m*/*z* 154.06 by HRESIMS at *m*/*z* 155.0698 [M + H]⁺ and *m*/*z* 177.0529 [M + Na]⁺. Interpretation of NMR and HRMS data (Figures S4–S8) suggested that the compound isolated was terrein, as previously reported [28,42].

### 3.4. Biological Activities

#### 3.4.1. Antimicrobial Activities of the Fungal Extracts

All 23 fungal isolates were cultured on a small scale, and the crude extracts of their broth and mycelia were tested for antimicrobial activity (against *P. aeruginosa* ATCC27853, *B. subtilis* ATCC6633, *S. aureus* ATCC25923 and *C. albicans* ATCC76615) using the agar diffusion assay [65]. Fungal isolate extracts displayed different levels of antimicrobial activities against at least one pathogen (Table 1). It is worth pointing out that the extracts of most fungal strains displayed exceptionally high antibacterial activities against *P. aeruginosa* and *S. aureus* (inhibition diameters more than 15 mm). Amongst these, the extract of fungal strain S013 exhibited high activity against *C. albicans*, while other extracts were inactive (Table 1).

From our investigation in this study, we found that extracts of some fungal broths and/or mycelia have antimicrobial effects and that different fungal strains could secrete intracellular and extracellular bioactive metabolic products [66]. Because of the efficacy of these extracts against *P. aeruginosa* and *S. aureus*, these extracts might have the potential to serve as drug leads to treat a wide variety of diseases. The results of the antimicrobial assay revealed that fungi derived from the marine source isolated in this study might be a prolific source of active compounds, which may hold potential as antibacterial and antifungal natural compounds.

#### 3.4.2. Antiproliferative Activities of the Fungal Extracts

All 23 fungal isolates were cultured on a small scale, and the crude extracts of their fungal broth and mycelia were tested for antiproliferative activity against MCF-7, HepG2, and HCT-116 cell lines using the SRB-U assay [67]. The extracts showed variable antiproliferative activity against the cell lines under investigation. Amongst these, extracts of S004, S006, S016, S017, and S020 presented the most promising antiproliferative profile (IC_50_ values of < 50 µg/mL) (Table 2). The present study revealed the diversity of the antiproliferative potential of marine fungal extracts and, hence, demonstrated their strong potential to produce cytotoxic compounds. Marine fungi and their purified extracts have been shown to be good producers of antiproliferative and cytotoxic compounds. This property could be attributed to different classes of secondary metabolites, as reported previously [20,25,26,27,68,69,70,71].

#### 3.4.3. Cytotoxic Activity of the Isolated Compound Terrein

The cytotoxic effect of terrein (Table 4) against HCT-116 and HepG2 cancer cell lines (concentration range 0.01–100 μM) was assessed using the SRB-U assay [67]. The compound displayed strong antiproliferative activity against the two cell lines under investigation, with IC_50_ values of 12.13 μM and 22.53 μM for HCT-116 and HepG2 cells, respectively. Doxorubicin was used as a standard cytotoxic control.

## 4. Conclusions

In conclusion, a total of 23 symbiotic fungi distributed among 3 genera were identified and isolated from marine sponges and sediment collected off Obhur (Saudi Arabia). The biological screening of small-scale cultures of these fungi yielded several target fungal strains which produced secondary bioactive metabolites. Amongst these isolates, the chromatographic separation of the crude extract of *A. terreus* strain S020 by silica gel column chromatography led to the isolation of pure crystalline terrain, with a high yield of 537.26 ± 23.42 g/kg dried crude extract; this represents the highest fermentation production of terrein to date. The chemical structure was elucidated on the basis of HRMS, 1D, and 2D NMR spectroscopic analyses, and by comparison with reported data. The isolated compound, terrein, showed strong cytotoxic activity against colorectal carcinoma cells and hepatocellular carcinoma cells, with IC_50_ values of 12.13 and 22.53 µM, respectively. Our study contributes to the understanding of fungal diversity and provides the basis for future investigations of these symbionts with respect to identifying and purifying the bioactive leads in these fungal extracts. This study also describes an efficient approach for producing bioactive terrein in a very high yield. Currently, there is great demand for the development of new drugs to combat the emergence of bacterial resistance to traditional antibiotics.

## Figures and Tables

**Table 1:** Antimicrobial activities of isolated fungal strains’ (S001–S023) crude extracts of both fermentation broth and mycelia.

Marine. Host	Fungal Strain		Culture Media	*Pseudomonas aeruginosa*		*Bacillus subtilis*		*Staphylococcus aureus*		*Candida albicans*	
	Genus	Strain		Broth	Mycelia	Broth	Mycelia	Broth	Mycelia	Broth	Mycelia
*Stylissa carteri*	*Penicillium*	S001	CZYB	−	+++	−	−	++++	++++	−	−
	*Aspergillus*	S002	CZYB	−	+++	−	−	++++	++++	−	−
Sediment	*Penicillium*	S003	CZYB	−	++++	−	−	++++	+++	−	−
	*Aspergillus*	S004	CZYB	−	++++	−	−	−	+++	−	−
	*Aspergillus*	S005	CZYB	+++	++++	−	−	++++	++++	−	−
	*Aspergillus*	S006	CZYB	−	++++	−	−	−	++++	−	−
	*Aspergillus*	S007	CZYB	−	++++	−	−	−	++++	−	−
	*Pleosporaceae*	S008	CZYB	−	++++	−	−	−	++++	−	−
*Hyrtios erectus*	*Aspergillus*	S009	MEB	−	−	−	−	−	+++	−	−
	*Penicillium*	S010	MEB	−	−	−	−	−	+++	−	−
	*Penicillium*	S011	MEB	−	+++	−	−	−	++++	−	−
Sediment	*Aspergillus*	S012	MEB	−	++++	+++	−	+++	++++	−	−
Sediment	*Aspergillus*	S013	MEB	−	++++	−	−	−	++++	++++	−
*H*. *erectus*	*Penicillium*	S014	SDB	−	+++	−	−	+++	+++	−	−
*S*. *carteri*	*Penicillium*	S015	SDB	++++	+++	−	−	++++	++++	−	−
Deep Sea	*Aspergillus*	S016	SDB	−	++++	+++	−	−	++++	−	−
	*Aspergillus*	S017	SDB	+++	++++	+++	−	+++	++++	−	−
	*Aspergillus*	S018	SDB	−	++++	−	−	−	++++	−	−
Sediment	*Aspergillus*	S019	SDB	−	+++	+++	−	+++	++++	−	−
	*Aspergillus*	S020	SDB	−	++++	−	−	−	++++	−	−
	*Aspergillus*	S021	SDB	−	++++	−	−	+++	++++	−	−
	*Aspergillus*	S022	SDB	+++	+++	−	−	+++	+++	−	−
	*Aspergillus*	S023	SDB	−	+++	−	−	+++	+++	−	−

Sabouraud dextrose broth (SDB); malt extract broth (MEB); Czapek–Dox broth (CZYB). Extracts tested at concentrations of 2 mg/mL; inhibition zone in mm including disc. Inhibition diameters were used to describe the groups of microbial growth inhibition: growth inhibition diameter more than 15 mm (++++); between 15 and 10 mm (+++); and less than 7 mm (+); no inhibition noticed (−).

**Table 2:** In vitro antiproliferative activities (IC_50_, μg/mL) of isolated fungal strains’ (S001–S023) crude extracts of both fermentation broth and mycelia against human carcinoma cells (MCF-7, HCT-116, and HepG2).

Marine Host	Fungal Strain		Culture Media	Cell Lines					
	Genus	Strain		Breast MCF-7		Hepatocellular HepG2		Colorectal HCT-116	
				Broth	Mycelia	Broth	Mycelia	Broth	Mycelia
*S. carteri*	*Penicillium*	S001	CZYB	75.44	˃100	79.26	˃100	˃100	92.60
	*Aspergillus*	S002	CZYB	73.28	˃100	61.96	˃100	˃100	48.00
Sediment	*Penicillium*	S003	CZYB	55.16	˃100	48.96	˃100	˃100	68.40
	*Aspergillus*	S004	CZYB	22.72	81.95	13.55	˃100	42.75	51.40
	*Aspergillus*	S005	CZYB	70.29	˃100	60.22	˃100	˃100	70.66
	*Aspergillus*	S006	CZYB	34.27	92.15	40.20	˃100	62.20	˃100
	*Aspergillus*	S007	CZYB	60.50	˃100	65.63	˃100	87.00	80.00
	*Pleosporaceae*	S008	CZYB	92.00	˃100	˃100	˃100	˃100	˃100
*H*. *erectus*	*Aspergillus*	S009	MEB	˃100	˃100	˃100	˃100	˃100	˃100
	*Penicillium*	S010	MEB	73.60	˃100	˃100	˃100	˃100	˃100
	*Penicillium*	S011	MEB	57.67	˃100	83.20	˃100	˃100	˃100
Sediment	*Aspergillus*	S012	MEB	˃100	92.00	92.00	˃100	˃100	97.00
Sediment	*Aspergillus*	S013	MEB	39.00	79.92	54.81	86.76	73.60	92.60
*H*. *erectus*	*Penicillium*	S014	SDB	79.53	˃100	92.80	˃100	˃100	˃100
*S*. *carteri*	*Penicillium*	S015	SDB	64.70	˃100	66.26	˃100	74.20	15.00
Deep Sea	*Aspergillus*	S016	SDB	23.27	93.50	41.70	67.44	62.00	81.00
	*Aspergillus*	S017	SDB	42.26	˃100	54.40	99.08	53.16	˃100
	*Aspergillus*	S018	SDB	60.48	˃100	97.70	˃100	90.00	˃100
Sediment	*Aspergillus*	S019	SDB	51.00	˃100	86.72	˃100	49.80	˃100
	*Aspergillus*	S020	SDB	44.00	81.95	57.00	˃100	47.83	67.00
	*Aspergillus*	S021	SDB	89.93	68.15	63.29	˃100	74.60	89.66
	*Aspergillus*	S022	SDB	65.12	87.00	91.80	˃100	81.60	˃100
	*Aspergillus*	S023	SDB	˃100	˃100	˃100	˃100	˃100	˃100
	Doxorubicin			0.41 ± 0.1		0.85 ± 0.1		0.11 ± 0.04	

Doxorubicin positive cytotoxic control, presented as the mean ± SD; n = 3.

**Table 3:** Identification of the isolated fungal strains (S001–S023). The closest relatives to fungal strains according to a BLAST search are presented.

Isolate	Genus Identification	Sequence Length (bp)	Related Strain (BLAST)	Access No.	Similarity (%)
S001	*Penicillium* sp.	522	*Penicillium chrysogenum*	KU556301	99%
S002	*Aspergillus* sp.	490	*Aspergillus cristatus*	KT151565	98%
S003	*Penicillium* sp.	524	*P. chrysogenum*	KX266831	98%
S004	*Aspergillus* sp.	531	*Aspergillus niger*	KJ881377	95%
S005	*Aspergillus* sp.	530	*A. niger*	KU904480	96%
S006	*Aspergillus* sp.	532	*Aspergillus flavus*	HQ645940	99%
S007	*Aspergillus* sp.	530	*Aspergillus tubingensis*	KX015988	97%
S008	*Pleosporaceae* sp	505	*Pleosporaceae* sp.	KJ443264	97%
S009	*Aspergillus* sp.	629	*Aspergillus* sp.	HQ023227	98%
S010	*Penicillium* sp.	534	*Penicillium polonicum*	KF494148	99%
S011	*Penicillium* sp.	525	*P. polonicum*	KP794074	98%
S012	*Aspergillus* sp.	527	*Aspergillus* sp.	KX015994	97%
S013	*Aspergillus* sp.	502	*Aspergillus foveolatus*	KU866615	99%
S014	*Penicillium* sp.	519	*P.chrysogenum*	KX015998	98%
S015	*Penicillium* sp.	526	*P. chrysogenum*	KU743900	98%
S016	*Aspergillus* sp.	523	*A. flavus*	FJ216388	98%
S017	*Aspergillus* sp.	529	*A. flavus*	KX015986	95%
S018	*Aspergillus* sp.	644	*A. niger*	KT377250	99%
S019	*Aspergillus* sp.	438	*A. niger*	LC195003	98%
S020	*Aspergillus* sp.	538	*Aspergillus terreus*	KX694148	98%
S021	*Aspergillus* sp.	330	*Aspergillus* sp.	KX000269	94%
S022	*Aspergillus* sp.	536	*Aspergillus fumigatus*	KU743889	98%
S023	*Aspergillus* sp.	531	*Aspergillus* sp.	KU842426	99%

**Table 4:** In vitro cytotoxic activity (IC_50_, µM) of terrein against human carcinoma cells (HCT-116 and HepG2).

Cell Type	Cell Line	Doxorubicin	Terrein
Colorectal	HCT-116	0.11 ± 0.04	12.13
Hepatocellular	HepG2	0.85 ± 0.1	22.53

Doxorubicin positive cytotoxic control, presented as the mean ± SD; n = 3.

## Supplementary Materials

The following are available online at https://www.mdpi.com/2218-273X/9/9/480/s1, Figure S1: HPLC chromatograms of terrein (A) and *Aspergillus terreus* S020 extract (B), Figure S2: UV-Vis spectra (200-400 nm) of crude extract of *A. terreus* S020 and terrein, Figure S3: The standard curve for quantifying terrein, Figure S4 (a,b): HRESIMS spectrum of terrein, Figure S5: ESIMS spectrum of terrein, Figure S6: ^1^H-NMR spectrum of terrein (CD_3_OD), Figure S7: ^13^C-NMR spectrum of terrein (CD_3_OD), Figure S8: HMBC spectrum of terrein (CD_3_OD).
