# Supplementary material for: Large-Scale Production of Bioactive Terrein by Aspergillus terreus Strain S020 Isolated from the Saudi Coast of the Red Sea
Source: Biomolecules. 2019 Sep 12;9(9):480. doi: 10.3390/biom9090480 (PMC6769563; doi:10.3390/biom9090480)
Supplement: Supplementary file 1 [file biomolecules-09-00480-s001.pdf]

Large Scale Production of Bioactive Terrein by *Aspergillus terreus* Strain S020 Isolated from the Saudi Coast of the Red Sea

Hani Z. Asfour [1], Zuhier A. Awan [2], Alaa A. Bagalagel [3], Mahmoud A. Elfaky [4] Reda F. Abdelhameed [5] and Sameh S. Elhady [4,6,*]

[1] Department of Medical Microbiology and Parasitology, Faculty of Medicine, Princess Al-Jawhara Center of Excellence in Research of Hereditary Disorders, King Abdulaziz University, Jeddah 21589, Saudi Arabia; hasfour@kau.edu.sa
[2] Department of Clinical Biochemistry, Faculty of Medicine, King Abdulaziz University, Jeddah, 21589, Saudi Arabia; zawan@kau.edu.sa
[3] Department of Clinical Pharmacy, Faculty of Pharmacy, King Abdulaziz University, Jeddah 21589, Saudi Arabia; abagalagel@kau.edu.sa
[4] Department of Natural Products and Alternative Medicine, Faculty of Pharmacy, King Abdulaziz University, Jeddah 21589, Saudi Arabia; melfaky@kau.edu.sa, ssahmed@kau.edu.sa
[5] Department of Pharmacognosy, Faculty of Pharmacy, Suez Canal University, Ismailia 41522, Egypt; omarreda_70@yahoo.com
[6] Department of Pharmacognosy, Faculty of Pharmacy, Port Said University, Port Said 42526, Egypt

* Correspondence: ssahmed@kau.edu.sa; Tel.: +966-544-512-552; Fax: +966-269-516-96

Supplementary Data

1) **Figure S1.** HPLC chromatograms of terrein (A) and *Aspergillus terreus* S020 extract (B)
2) **Figure S2**. UV-Vis spectra (200-400 nm) of crude extract of *A. terreus* S020 and terrein.
3) **Figure S3**. The standard curve for quantifying terrein
4) **Figure S4 (a,b)**. HRESIMS spectrum of terrein
5) **Figure S5**. ESIMS spectrum of terrein
6) **Figure S6**. ^{1}H-NMR spectrum of terrein (CD_3OD)
7) **Figure S7**. ^{13}C-NMR spectrum of terrein (CD_3OD)
8) **Figure S8**. HMBC spectrum of terrein (CD_3OD)

General Experimental Procedures

Amplification of Fungal ITS-rDNA Fragments was done by Thermal cycler (TC-5000, Techine, USA). PCR products were detected by Horizontal Electrophoresis (HU 10, UK) using Gel Documentation System (G:Box, Syngene, UK). Sequence and Phylogenetic Analysis were obtained using DNA analyzer Applied Biosystems (Applied Biosystems, Inc., Foster City, CA, USA). Optical rotation was measured on the automatic high-speed laboratory polarimeter P3000 (A.KRUSS Optronic Gmbh, Hamburg, Germany). UV spectra were measured on a Hitachi 300 Spectrophotometer (Hitachi High- Technologies Corporation, Kyoto, Japan). High-resolution ESIMS data were recorded with an Ultra-High Resolution (UHR) TOF spectrometer (Impact, Bruker, Bremen, Germany). NMR spectra were obtained in CD_3OD on a Bruker Avance DRX 600-MHz spectrometer (Bruker, Bremen, Germany) at 600-MHz for ^1H NMR and 150 MHz for ^{13}C NMR. NMR chemical shifts were expressed in parts per million (ppm) referenced to residual CD_3OD solvent signals (δ_H 7.26 for ^1H and δ_C 77.0 for ^{13}C). Precoated SiO_2 60 F_{254} plates (Merck, Darmstadt, Germany) were used for TLC. SiO_2 (70–230 mesh, Merck, Darmstadt, Germany) was used for column chromatography. Separation on HPLC were performed using column (5 μm ZORBAX Eclipse XDB-C18, 250 × 4.6 mm, Agilent, Santa Clara, CA, USA).

Determination of terrein using HPLC

The content of terrein was determined using a The HPLC system was an Agilent 1200 series (Agilent Technologies, Germany) consisting of a solvent delivery module, a quaternary pump, an autosampler, a diode-array detector (DAD) and a column compartment. The control of the HPLC system and data processing were performed using ChemStation [Rev. B.01.03 SR2 (204)]. The chromatograms were detected by HPLC (Zorbax Eclipse XDP-C18 column, 5 μm, 250 × 4.6 mm (I.d), Agilent, Santa Clara, CA, USA) at 20% CH_3CN/H_2O, 1 mL/min flow rate and 281 nm UV detection). All determinations were performed at ambient temperature. The compound was confirmed by adding standard method and the retention time of 3.32 min from chromatograms by ultraviolet absorption at a wavelength of 281 nm. The injection volume was 5 μL. Each sample was repeated in triplicate. The calibration curve and method validation are described in **Figure S3** The content of **terrein** was calculated according to the calibration curve and expressed as g/Kg dry weight (dw).

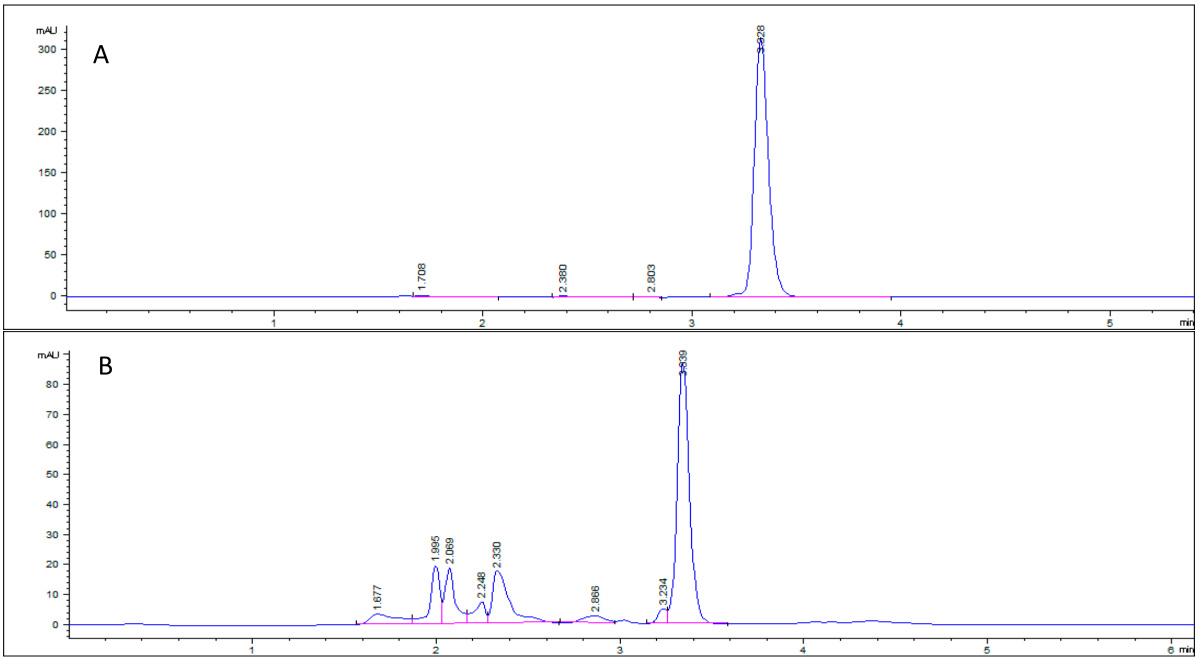

Figure S1. HPLC chromatograms of terrein (A) and *Aspergillus terreus* S020 extract (B)

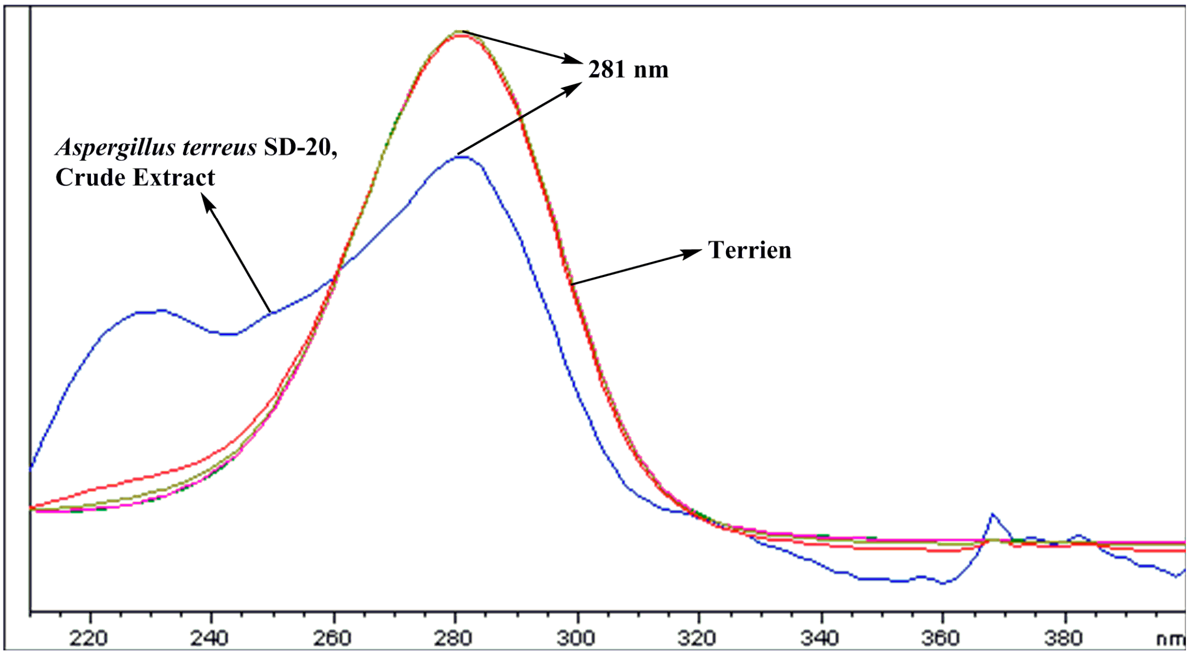

Figure S2. UV-Vis spectra (200-400 nm) of crude extract of *Aspergillus terreus* S020 and terrein.

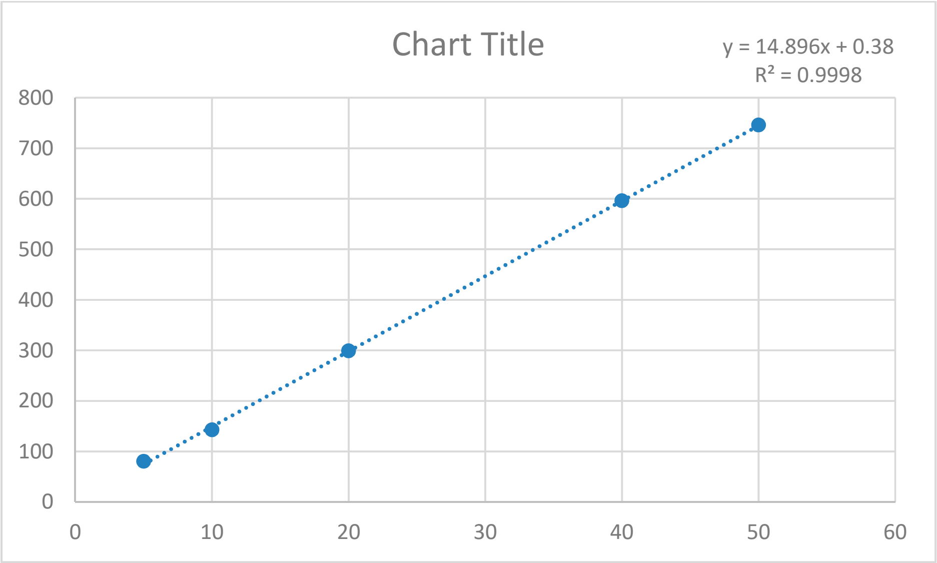

Figure S3. The standard curve for quantifying terrein

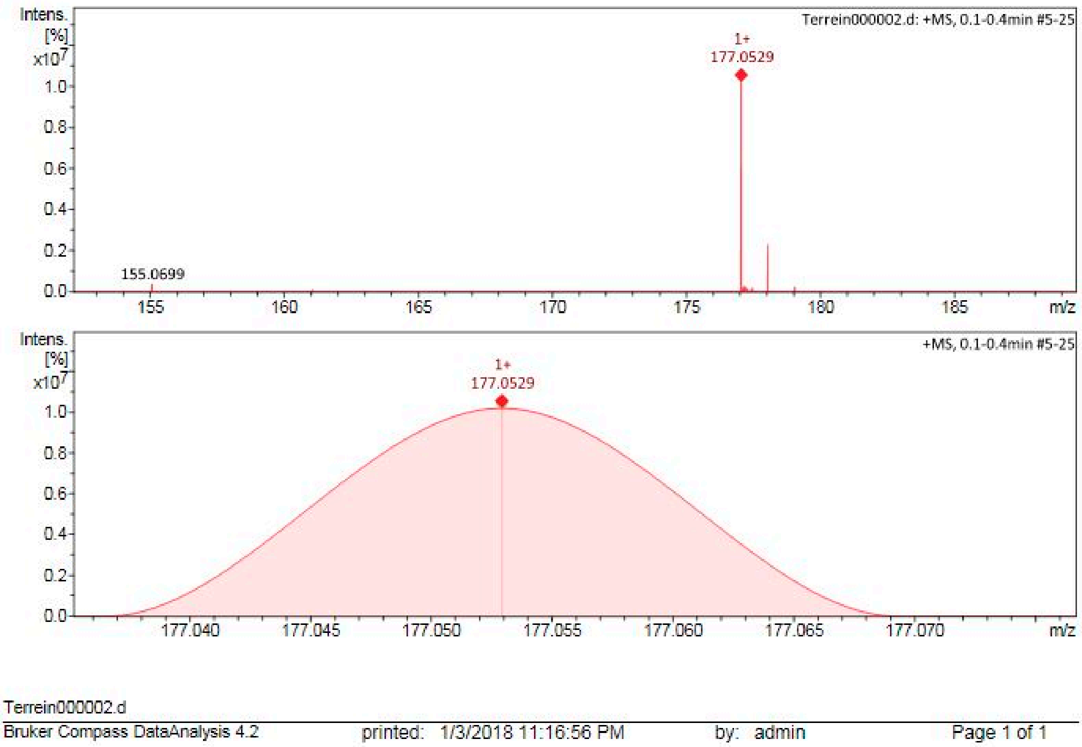

Figure S4-a. HRESIMS, spectrum of terrein

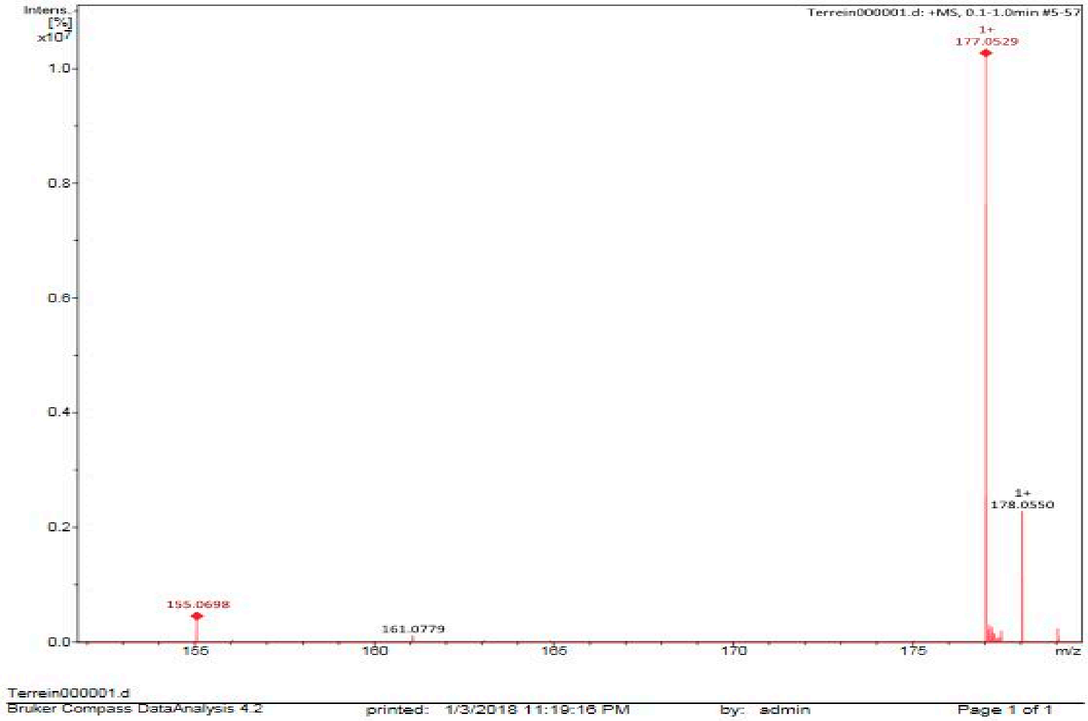

Figure S4-b. HRESIMS, spectrum of terrein

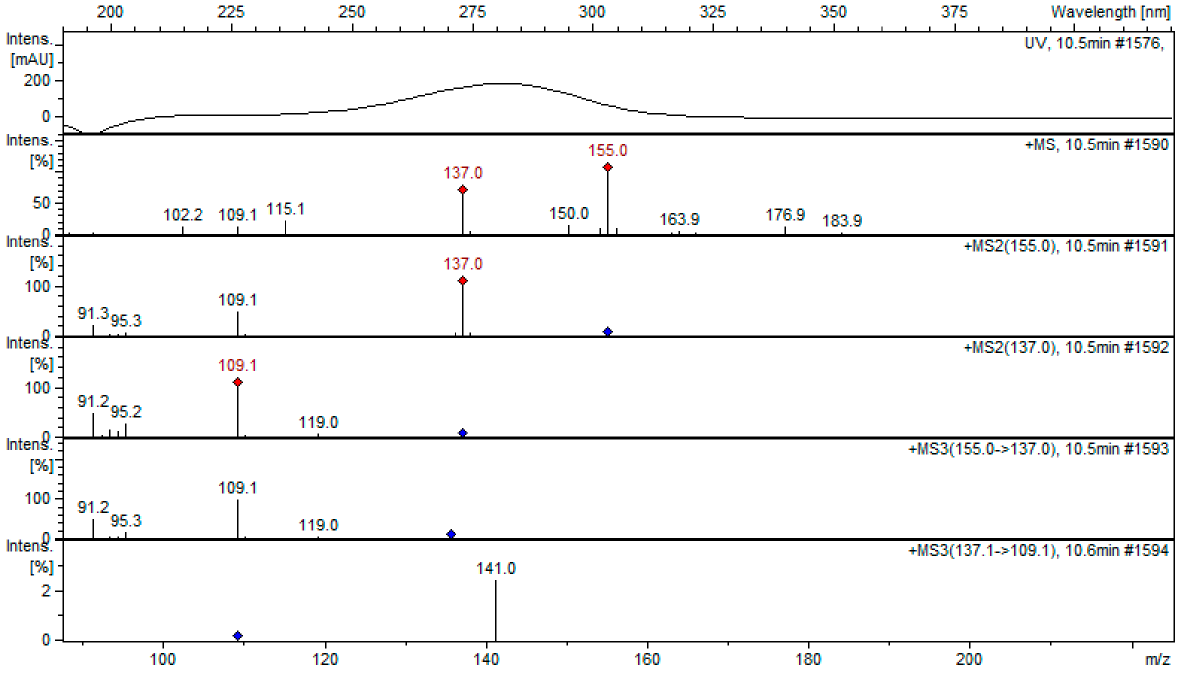

Figure S5. ESIMS spectrum of terrein

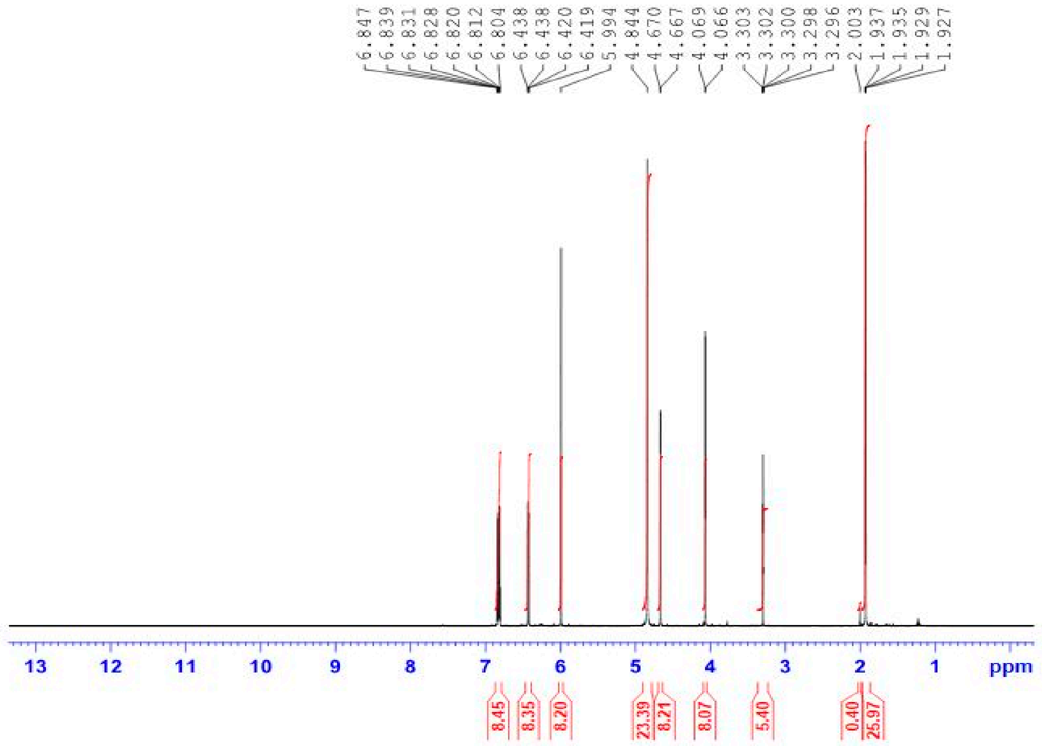

Figure S6. ^{1}H-NMR spectrum of terrein (CD_3OD)

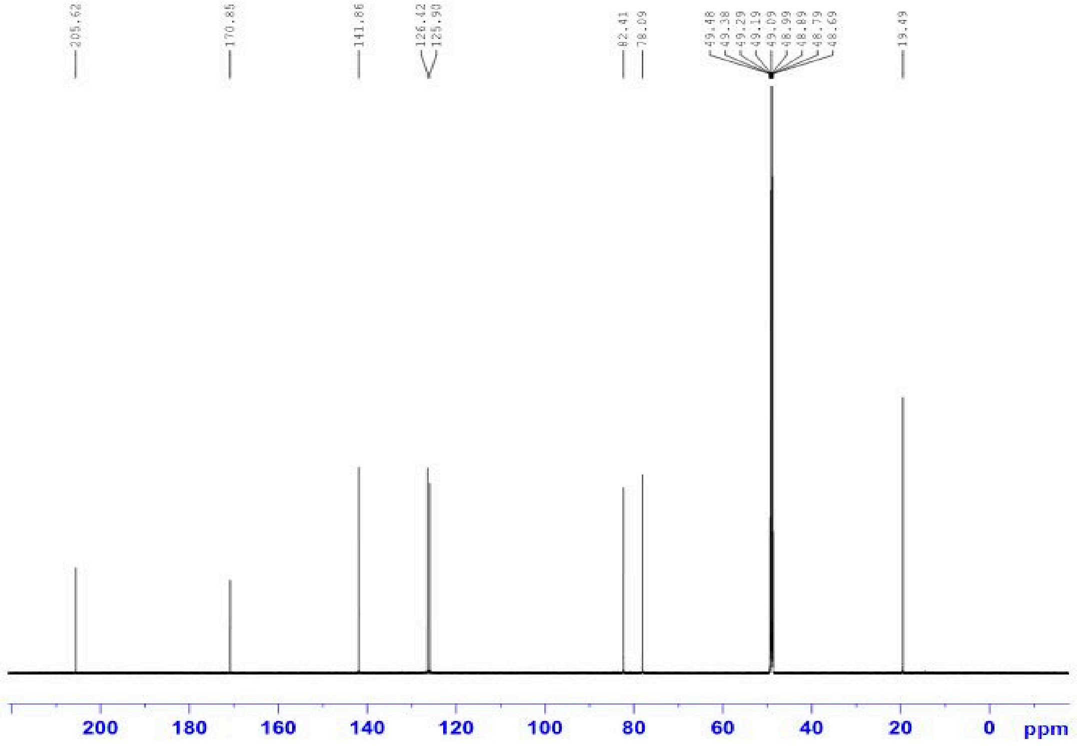

Figure S7. ^{13}C-NMR spectrum of terrein (CD_3OD)

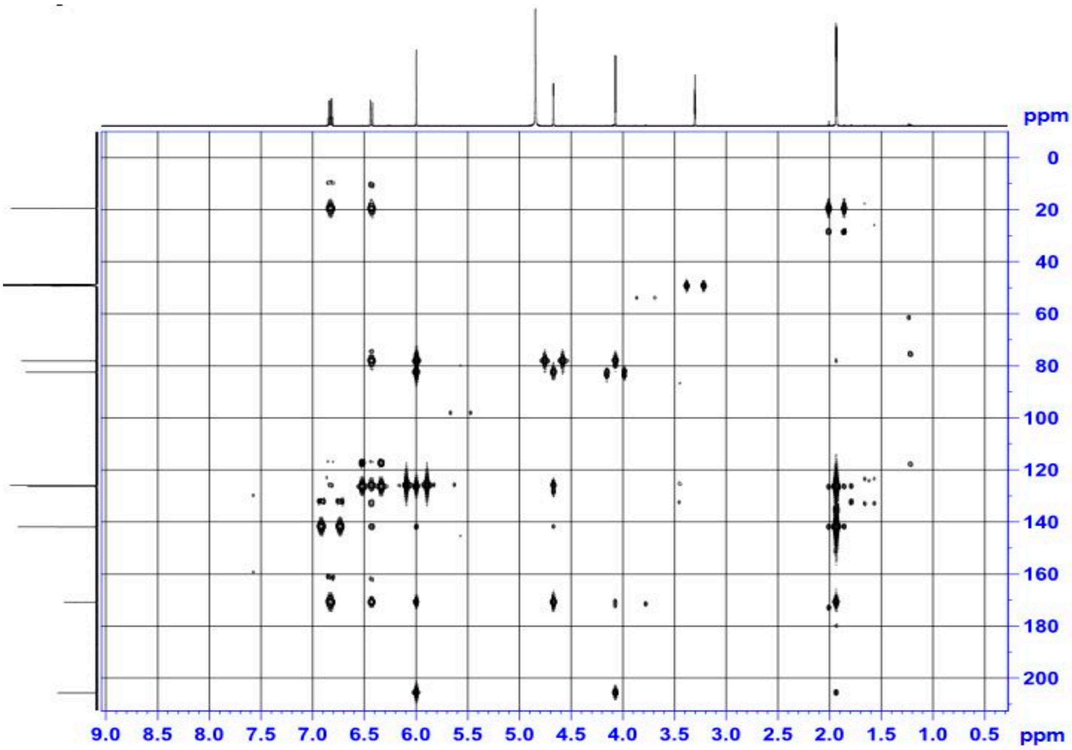

Figure S8. HMBC spectrum of terrein (CD_3OD)
